# Quantitative detection of fecal contamination with domestic poultry feces in environments in China

**DOI:** 10.1186/s13568-017-0379-0

**Published:** 2017-04-14

**Authors:** Fang-Fang Zhuang, Hu Li, Xin-Yuan Zhou, Yong-Guan Zhu, Jian-Qiang Su

**Affiliations:** 1 Key Lab of Urban Environment and Health, Institute of Urban Environment, Chinese Academy of Sciences, 1799 Jimei Road, Xiamen, 361021 China; 2 University of Chinese Academy of Sciences, Beijing, 100049 China

**Keywords:** Poultry, Source tracking, Pollution, Mitochondrial DNA, Quantitative PCR, Human impacts

## Abstract

Poultry are an important source of fecal contamination in environments. However, tools for detecting and tracking this fecal contamination are in the early stages of development. In practice, we have found that source tracking methods targeting the 16S rRNA genes of poultry-specific microbiota are not sufficiently sensitive. We therefore developed two quantitative PCR assays for detection of poultry fecal contamination, by targeting chicken and duck mitochondrial genes: NADH dehydrogenase subunit 5 (*ND5*) and cytochrome *b* (*cytb*). The sensitivity of both assays was 100% when tested on 50 chicken and duck fecal samples from 10 provinces of China. These assays were also tested in field samples, including soil and water collected adjacent to duck farms, and soils fertilized with chicken manure. Poultry mitochondrial DNA was detected in most of these samples, indicating that the assays are a robust method for monitoring environmental contamination with poultry feces. Complemented with existing indicators of fecal contamination, these markers should improve the efficiency and accuracy of microbial source tracking.

## Introduction

Animal and human feces carry many enteric pathogens, and when these contaminate water bodies, they pose a risk to human health (WHO Scientific group 2011; Harwood et al. 2014). Contamination with feces is a global public health issue from the perspective of the microbiological safety of water (Simpson et al. 2002; Blanch et al. 2006). However, detection and identification of pathogens is time-consuming and costly, due to the diversity and low concentrations of pathogens in environments (Field and Samadpour 2007). Instead, the presence of pathogens is often assumed based on detection of fecal indicator bacteria (FIB) including fecal coliforms and enterococci. These microorganisms are found in many animals and can survive and reproduce in diverse environments (Harwood et al. 2014). Thus, the source, and extent of pollution with FIB can be difficult to identify.

Domestic chickens and ducks are common around the world, and especially so in China. Commercial and backyard poultry farming both result in discharge of poultry feces into the environment, particularly after storms and floods. In addition, poultry manure is frequently applied to agricultural fields as fertilizer (Ryu et al. 2014). Poultry feces are known to carry human pathogens (McMurry et al. 1998; Hofacre et al. 2000), and consequently, attention needs to be paid to pollution with poultry feces. In particular, robust assays are needed to monitor environments in the interest of public health.

Microbial source tracking (MST) methods (Wiggins 1996; Parveen et al. 1997) use host-specific genetic markers as alternative indicators of fecal contamination from various sources (Scott et al. 2002). MST markers have been developed for identifying the source of fecal pollution by targeting the 16S rRNA genes and other genes of host-associated microbiota, or by targeting host-specific mitochondrial DNA (mtDNA).

MST methods for humans and other animals such as pigs, dogs, and cattle are well developed. In contrast, real-time PCR assays targeting domestic chickens and ducks are not well established. Poultry-specific source tracking markers have been developed, but these assays mostly target chicken (Schill and Mathes 2008; Gómez-Doñate et al. 2012; Kobayashi et al. 2013; Dancer et al. 2014; Ryu et al. 2014; Ohad et al. 2016). Further, 16S rRNA markers for ‘host-specific’ microorganisms often cross-react with the microbiota of other animals (Layton et al. 2006; Kildare et al. 2007; Marti et al. 2011; Ryu et al. 2012; Boehm et al. 2013; Green et al. 2014a). Functional genetic markers for host-specific microorganisms often have issues with sensitivity (Ebentier et al. 2013). In our hands, source tracking markers targeting enteric microbial genes of chicken and poultry showed low sensitivity and were not effective for detecting fecal contamination from poultry. Methods based on species-specific eukaryotic mtDNA markers might show more promise for fecal source tracking (Caldwell et al. 2011).

The objective of this study was to develop real-time PCR assays that targeted chicken and duck mtDNA with high sensitivity and specificity. Two novel assays were developed targeting poultry mitochondrial genes: NADH dehydrogenase subunit 5 (*ND5*) and cytochrome *b* (*cytb*). We test 173 fecal samples from 12 host species to test the performance of the two assays. Subsequently, the assays were applied to environmental soil and water samples.

## Materials and methods

### Sample collection

A total of 173 fecal samples were collected between August 2015 and July 2016 from 14 provinces of China, including Inner Mongolia, Beijing, Hebei, Tianjin, Shandong, Shanxi, Jiangsu, Zhejiang, Jiangxi, Fujian, Guangdong, Tibet, Hong Kong, and Hainan Province. Fecal samples were from a variety of host animals, including chickens (n = 38), ducks (n = 9), poultry (chicken and duck composite fecal samples, n = 3), humans (n = 25), pigs (n = 17), dogs (n = 23), cattle (n = 17), sheep (n = 16), cats (n = 5), horses (n = 8), deer (n = 1), geese (n = 2), and rabbits (n = 9). Fresh feces were frozen and transported on ice to the laboratory within 3 days. All samples were stored at −20 °C until use.

Soil and water samples from eight duck farms in Yizhou of Guangxi Province and one duck farm in Longyan of Fujian Province were collected in September 2016. Samples were immediately frozen at −20 °C and transported on ice to the laboratory within 24 h.

Soils fertilized with chicken manure (CM) were collected from a long-term experiment station of the Chinese Academy of Agricultural Sciences (CAAS) in Dezhou, Shandong Province. A total of 24 samples from eight treatments in triplicate were collected in August 2015, about ten months after the application of chicken manure, urea or sewage sludge. One treatment was fertilized with chicken manure, and the other seven treatments were fertilized with urea or sewage sludge. The soils were immediately frozen on dry ice, transported to the laboratory within 24 h and stored at −80 °C until analysis. Details on the design of this field experiment are described by Chen et al. (2016).

### Sample pretreatment and DNA extraction

Water samples (0.5 L) were filtered through 0.22-μm mixed cellulose ester membrane, which were cut into small pieces using aseptic scissors and placed into Lysing Matrix tubes provided in the FastDNA^®^ SPIN Kit for Soil (MP Biomedical, Santa Ana, California, USA). Total DNA of soil and water samples was extracted according to the manufacturer’s protocol. Total DNA of fecal samples was extracted from 0.5 g frozen samples using FastDNA^®^ SPIN Kit for Feces (MP Biomedical, Santa Ana, California, USA), following the manufacturer’s instructions. The quality and quantity of DNA were analyzed by spectrophotometry using NanoDrop ND-1000 (Nanodrop, USA). The extracted DNA was stored at −20 °C.

### Primer and probe design

**Fig. 1:**
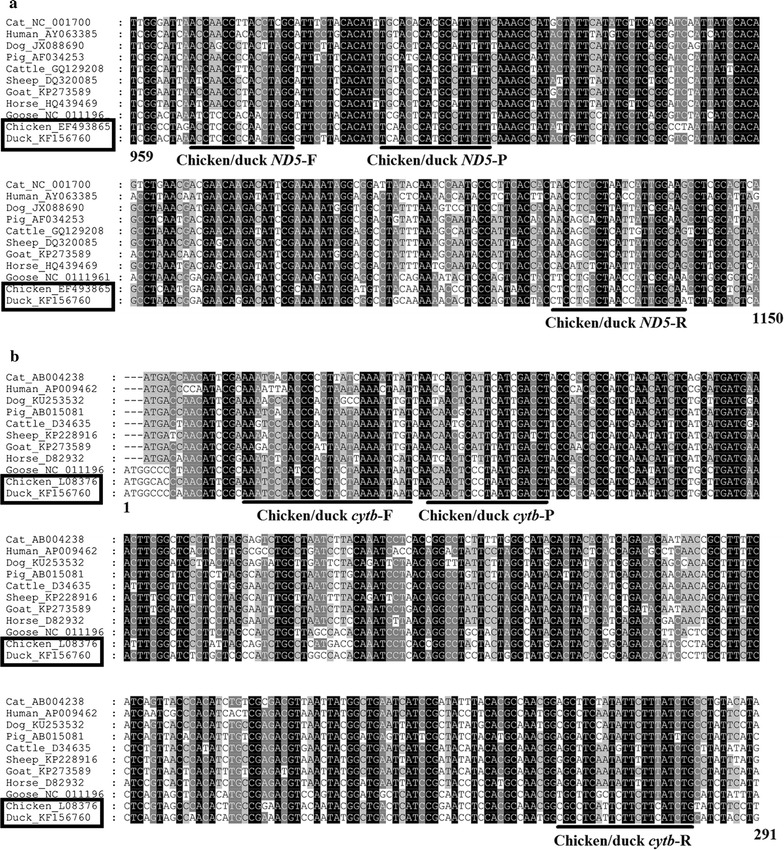
Alignments of mtDNA *ND5* and *cytb* gene from cat, human, dog, pig, cattle, sheep, goat, horse, goose, chicken, and duck. Binding sites of primers and probes to the mtDNA *ND5* and *cytb* gene sequences are indicated. **a** mtDNA *ND5* assay and **b** mtDNA *cytb* assay

Multiple alignments of the target host mtDNA (Fig. 1) were performed using the program Clustal X (Larkin et al. 2007). Nucleotide sequences were retrieved from NCBI GenBank™ under accession numbers: chicken (L08376 and EF493865), duck (KF156760), human (AP009462 and AY063385), cattle (D34635, and GQ129208), dog (KU253532 and JX088690), pig (AB015081 and AF034253), sheep (KP228916 and DQ320085), goat (KP273589), cat (AB004238 and NC_001700), horse (D82932 and HQ439469), goose (NC_011196). New primers and probes for mitochondrial genes NADH dehydrogenase subunit 5 (*ND5*) and cytochrome *b* (*cytb*) for chicken and duck (Table 1) were designed manually, and then confirmed with OligoAnalyzer software from IDT (Owczarzy et al. 2008). Minor groove binder (MGB) probes were designed for chicken and duck *ND5*, and chicken and duck *cytb* markers. The 6-carboxy-fluorescein (FAM) was conjugated at the 5′ ends of the MGB probes, and a non-fluorescent quencher (NFQ) was conjugated at the 3′ ends. Primers were synthesized by Invitrogen (Carlsbad, CA, USA) and TaqMan probes were synthesized by Applied Biosystems (Foster City, CA, USA). All oligonucleotides were reconstituted in TE buffer (pH 7.5) and stored at −20 °C prior to use.

**Table 1:** Oligonucleotide sequences for quantitative PCR

Primer or probe	Oligonucleotide sequence (5′–3′)	Tm (°C)	Amplicon size (bp)
Chicken and duck *ND5*-F	ACCTCCCCCAACTAGC	53.6	172
Chicken and duck *ND5*-R	TTGCCAATGGTTAGGCAGGAG	57.7	
Chicken and duck *ND5*-P	(FAM)TCAACCCATGCCTTCTT(NFQ-MGB)	61.1	
Chicken and duck *cytb*-F	AAATCCCACCCCCTACTAAAAATAAT	54.3	263
Chicken and duck *cytb*-R	CAGATGAAGAAGAATGAGGCG	53.4	
Chicken and duck *cytb*-P	(FAM)ACAACTCCCTAATCGACCT(NFQ-MGB)	62.7	

### Standard curve generation

To make standard plasmids, the purified chicken and duck *ND5* and *cytb* gene products of traditional PCR were cloned using the pMD19-T vector (Takara, Bio Inc., Shiga, Japan), and were then sequenced. The plasmids with correct target genes were extracted using TIANprep MINI plasmid kit (Tiangen, Beijing, China). Standard curves of chicken and duck *ND5* and *cytb* were generated using tenfold serial dilutions (9.70 × 10^7^ − 10^0^ and 1.08 × 10^8^ − 10^1^ copies per μL respectively) of standard plasmids containing chicken and duck *ND5* or *cytb* genes.

### Quantitative PCR

Chicken and duck *ND5* and *cytb* gene abundances were determined by qPCR using the LightCycler 480 real-time PCR detection system (Roche480, USA). A 20 μL aliquot of qPCR mixture contained 10 μL of 2× TaqMan^®^ Gene Expression Master Mix (Applied Biosystems, Foster City, CA, USA), 0.9 μM each of forward and reverse primers, 0.25 μM of TaqMan probe, 1 mg mL^−1^ of bovine serum albumin (BSA, Sigma, Steinheim, Germany), 20–100 ng template DNA, and sterile ddH_2_O. The qPCR was conducted with the following conditions: 50 °C for 2 min (activation of the uracil-N-glycosylase) and 95 °C for 10 min (activation of the AmpliTaq Gold DNA polymerase), followed by 40 cycles of denaturation at 95 °C for 15 s and annealing and extension at 60 °C for 1 min. No template controls were used for all assays. All samples, standards, and controls were run in triplicate. A sample was classified as quantifiable if two or more replicates were above the limits of detection (LOD). Only amplification efficiencies between 85 and 115% were considered as acceptable for quantification.

### Statistical analysis

Amplification efficiency (*E*) was determined using the slope of the standard curve, as follows: *E* = 10^(−1/slope)^ − 1. The LOD was defined as the lowest concentration of the marker within the linear range of a quantification curve. Sensitivity was calculated as the fraction of actual positive [true positive (TP)] host samples divided by all expected positive hosts, including both false negative (FN) and TP, as follows: Sensitivity = TP/(FN + TP). Specificity was calculated as the fraction of actual negative [true negative (TN)] host samples divided by all expected negative hosts including both unexpected positive [false positive (FP)] and TN, as follows: Specificity = TN/(FP + TN).

## Results

### Design of chicken and duck-specific mtDNA *ND5* and *cytb* genetic markers

Multiple alignments of mtDNA *ND5* and *cytb* gene from cat, human, dog, pig, cattle, sheep, goat, horse, goose, chicken, and duck were performed using Clustal X. We designed two assays targeting chicken and duck mtDNA *ND5* and *cytb* gene, the expected amplicon sizes being 172 and 263 bp, respectively (Table 1). Based on the multiple alignments, chicken and duck *ND5* and *cytb* could be readily differentiated from the other selected hosts (Fig. 1).

### qPCR assays

Standard curves were generated using tenfold serial dilutions of chicken and duck *ND5* and *cytb* gene standard plasmids to determine the linear range, amplification efficiencies, and LOD. Linear range amplification was between 9.70 × 10^0^ − 10^7^ copies per μL for the *ND5* assay and 1.08 × 10^1^ − 10^8^ copies per μL for the *cytb* assay. The linear regression correlation coefficients (R^2^) were 0.9993 for the *ND5* assay and 0.9989 for the *cytb* assay. Amplification efficiency was 0.997 for the *ND5* assay and 0.964 for the *cytb* assay, both of which were within the 0.9–1.1 tolerance. The LOD was 0.97 and 1.08 gene copies per reaction for the *ND5* and *cytb* assays, respectively. Sensitivity and specificity of the assays were tested using total DNA extracted from 173 fecal samples of 12 common animals (Table 2).

**Table 2:** qPCR results of fecal samples from various host

Fecal samples	Number of samples	*ND5* assay		*Cytb* assay	
		Positive	Concentration^a^	Positive	Concentration^a^
Chicken and duck	50	100% (50/50)	7.8 ± 0.8	100% (50/50)	7.4 ± 0.8
Human	25	40% (10/25)	5.6 ± 0.4	28% (7/25)	5.2 ± 0.3
Pig	17	0%		0%	
Dog	23	17.3% (4/23)	5.2 ± 0.8	13.0% (3/23)	4.9 ± 0.6
Cattle	17	17.6% (3/17)	5.9 ± 1.4	11.8% (2/17)	6.1 ± 1.4
Sheep	16	0%		0%	
Cat	5	0%		0%	
Horse	8	12.5% (1/8)	5.5	12.5% (1/8)	5.2
Deer	1	0%		0%	
Goose	2	50% (1/2)	6.1	50% (1/2)	5.8
Rabbit	9	0%		0%	

^a^Concentrations are expressed as log10 copies per g dry feces

The sensitivity of the *ND5* assay was 100%, with 50/50 samples generated strong positive results. The quantified values (average ± standard deviation) of these samples were 7.8 ± 0.8 log_10_ copies per g dry feces. Positive results were also obtained from some human, dog, cattle, horse, and goose feces samples, but the average quantified values for these samples were all one to two orders of magnitude lower than those for chicken and duck samples (Table 2). If these lower quantification values are included as genuine positives, the specificity of the *ND5* assay was 84.6%.

The sensitivity of the *cytb* assay was also 100% (50/50 positives). The quantified values for these samples were 7.4 ± 0.8 log_10_ copies per g dry feces. Positive results were also obtained from some human, dog, cattle, horse, and goose feces, and these were the same samples as were found positive in the *ND5* assay. This strongly suggests there was minor contamination with poultry DNA in at least some of the samples. Again, the average quantified values for these samples were all one to two orders of magnitude below that of the genuine poultry samples. The specificity of *cytb* assay was 89.8% if these low concentration positives are included in the calculation (Table 2).

### Application of qPCR assays on field samples

We then applied the qPCR assays to environmental soil and water samples from nine duck farms and soils fertilized with chicken manure. All water samples tested positive using the *ND5* assay, with copy numbers ranging from 2.8 log_10_ copies per 100 mL to 6.4 log_10_ copies per 100 mL water (Table 3). The *cytb* assay gave similar results, except for one Duck farm (YZ4), whose value was below the LOD. This sample had the lowest copy number using *ND5* assay (Table 3).

**Table 3:** qPCR assays conducted on field samples, including soils amended with chicken manure (DZ-1CM—Dezhou), soil and water from duck farms in Longyan (LY) and Yizhou (YZ)

Sample	*ND5* assay^a^	*Cytb* assay^a^
DZ-1CM^b^	4.3	4.0
Duck farm water-LY	6.4	5.9
Duck farm water-YZ1	3.6	3.1
Duck farm water-YZ2	4.0	3.2
Duck farm water-YZ3	4.4	3.8
Duck farm water-YZ4	2.8	Not detected
Duck farm water-YZ5	4.6	4.3
Duck farm water-YZ6	3.8	3.3
Duck farm water-YZ7	4.2	4.0
Duck farm water-YZ8	3.5	3.0
Duck farm soil-LY	8.1	8.0
Duck farm soil-YZ1	5.1	4.6
Duck farm soil-YZ2	Not detected	Not detected
Duck farm soil-YZ3	Not detected	Not detected
Duck farm soil-YZ4	Not detected	Not detected
Duck farm soil-YZ5	Not detected	Not detected
Duck farm soil-YZ6	Not detected	Not detected
Duck farm soil-YZ7	Not detected	Not detected
Duck farm soil-YZ8	Not detected	Not detected

^a^Concentrations are expressed in log10 copies per g dry soil or log10 copies per 100 mL water for environmental water samples

^b^qPCR results of the other seven treatments from Dezhou, including control and field soil amended with urea or sewage sludge, were below the LOD and not shown in the table

One Yizhou soil sample from eight was positive with the *ND5* and *cytb* assays, returning gene copy numbers of 5.1 and 4.6 log_10_ copies per g dry soil, respectively. The Longyan soil sample was positive at the high concentrations of 8.1 log_10_ *ND5* gene copies per g dry soil and 8.0 log_10_ *cytb* gene copies per g dry soil.

Both *ND5* and *cytb* assays target mtDNA, and because the number of mtDNA molecules remains constant, the two assays should ideally return identical values for copy number in any particular sample. The values returned from analysis of feces, water and soil samples are in general agreement between the assays (Pearson correlation r = 0.9828, P < 0.001), with the *ND5* assay being consistently more sensitive (Tables 2, 3).

Soil samples amended with chicken manure tested positive using both *ND5* and *cytb* assays, returning concentrations of 4.3 and 4.0 log_10_ copies per g dry soil respectively. This is despite sampling being undertaken 10 months after the application of manure. Seven other treatments taken from different plots in the same long term field experiment were below the LOD (data not shown). These plots had been amended with other fertilizers such as urea and sewage sludge.

## Discussion

In this study, qPCR assays were developed to targeting chicken and duck mitochondrial *ND5* and *cytb*, with the aim of applying these for source tracking of poultry fecal contamination. Both assays were host-sensitive and host-specific when tested with fecal samples, and were robust enough to be used for analyzing field samples of soil and water.

Using both assays in tandem allowed us to compare their efficiency, since they both target the same molecule, mtDNA. In general, these copy number estimates agreed between the assays, although the *ND5* assay consistently returned slightly higher copy estimates (Tables 2, 3). The amplification efficiency of the *ND5* assay may have been higher. Because the size of *ND5* amplicon (172 bp) was shorter than that of *cytb* amplicon (263 bp).

We tested 173 fecal samples from 12 hosts (Table 2) from a large geographical range in China. Both *ND5* and *cytb* assays exhibited cross-reactions to some human, dog, cattle, horse and goose fecal samples. In general, the same samples were responsible for unexpected positives in both assays, suggesting that minor contamination might be responsible. In the case of human and dog fecal samples, this contamination might have arisen from recent consumption of poultry products. Positive signals for beef mtDNA *ND5* were obtained from two out of four human volunteers who consumed beef within 24 h of sampling (Caldwell et al. 2007). In the same study, there was no carry-over signal from consumption of pork. Further studies are needed to confirm whether human consumption of poultry products results in poultry mtDNA being present in feces. We also detected a small number of unexpected positive signals from cattle, horse, and goose fecal samples, which might simply be from environmental cross-contamination of samples. In any case, these unexpected positives could be easily distinguished from genuine positive samples because copy number estimates were generally one to two orders of magnitude lower (Table 2).

In preliminary experiments, we tested five reported chicken and poultry markers, including CL-TaqMan (Ryu et al. 2014), PLprobe (Gómez-Doñate et al. 2012), Chicken/Duck-Bac (Kobayashi et al. 2013), Duck-Bac (Kobayashi et al. 2013), and Av43 (Ohad et al. 2016), which target host-associated *Brevibacterium* sp., *Bifidobacterium*, *Bacteroides* spp., *Bacteroides* spp., and *Firmicutes* 16S rRNA gene, respectively. It has been reported that CL-TaqMan, Chicken/Duck-Bac, and Duck-Bac assays showed relatively low sensitivity and specificity (Kobayashi et al. 2013; Ryu et al. 2014). Our results agreed with this conclusion. The sensitivity of CL-TaqMan, PLprobe, Chicken/Duck-Bac, and Duck-Bac assays were all less than 50%. PLprobe, Chicken/Duck-Bac, and Av43 assays exhibited cross-reactions with dog, cat, sheep, cow or pig feces (data not shown). The low sensitivity of markers might be caused by the low abundance of targeted microorganisms in the host feces, and microbiota might vary significantly with diet (Turnbaugh et al. 2009; Muegge et al. 2011; Wu et al. 2011; David et al. 2014).

Currently, most MST methods rely on fecal indicator microorganisms or use molecular methods that require extensive data collection before hosts can be reliably differentiated (Green et al. 2014b; McLellan and Eren 2014; Ohad et al. 2016). Our real-time PCR assays targeting poultry mtDNA genes can overcome the above disadvantages. Firstly, mtDNA is remarkable for its species-specificity and low intra-species variability (Moritz et al. 1987). It can thus be used to identify animal species directly rather than rely on microbial species which may, or may not occur in the animal host (Caldwell et al. 2007). Crucially, there are large numbers of epithelial cells shed in feces (Iyengar et al. 1991) and mtDNA has many gene copies per cell (Andreasson et al. 2002). Therefore, mtDNA genes are likely to generate robust signals, simply because of their abundance (Martellini et al. 2005). Thirdly, high abundance means a long half-life, and potential for persistence in environmental compartments. Several groups have reported mtDNA from human and dog feces can persist in water for 15–29 days (Martellini et al. 2005; Tambalo et al. 2012), and mtDNA markers can survive for relatively long time in water bodies (He et al. 2015). Finally, the mtDNA of most common species have been fully sequenced and are freely available.

Fecal contamination of water bodies is a common environmental problem (WHO Scientific group 2000; McLellan and Eren 2014). The qPCR assays we developed here were sensitive, having a detection limit of 0.97 and 1.08 gene copies per reaction for the *ND5* and *cytb* assays, respectively (Table 2). This made it possible to readily identify poultry fecal contamination in environmental samples. We applied the *ND5* and *cytb* assays to environmental soil and water samples from nine duck farms. All water samples were positive with the exception of one *cytb* assay. Two soil samples collected near duck farms detected poultry fecal pollution. For most soil samples, although the concentration of total DNA was high (data not shown), no poultry fecal contamination was detected. Density of farm is likely one of the factors for low detection rate. Another reason may due to the random distribution of duck feces in farm soil. Detection of fecal contamination in soil is more difficult to achieve due to the heterogeneity of soil, limitation in sample collection, and DNA extraction, since only a small fraction (0.5 g in this study) of soil was subjected to DNA extraction and PCR amplification. In addition, contaminants in water are more mobilized than that in soil, leading to rapid spread in water bodies.

To test if this was because the mtDNA degraded more rapidly in soil, we tested soil where chicken manure had been applied. Samples taken ten months after the application of chicken manure were still positive in both assays, suggesting that the *ND5* and *cytb* assays are robust enough to be used on field samples.

In conclusion, we developed qPCR assays targeting the chicken and duck mitochondrial *ND5* and *cytb* genes. Both assays were sensitive and specific, as tested against 173 fecal samples from diverse animals. We also tested the assays on field samples, detecting poultry mtDNA soil and water samples adjacent to duck farms, and soil amended with chicken manure. Further studies are needed to investigate the low levels of unexpected positive reactions to some other fecal samples, in particular to test whether consumption of poultry results in mtDNA being present in human feces. Overall, the assays should be a useful addition to existing methods for detecting domestic chicken and duck fecal pollution.

## Abbreviations

*ND5*: NADH dehydrogenase subunit 5
*cytb*: cytochrome *b*
FIB: fecal indicator bacteria
MST: microbial source tracking
mtDNA: mitochondrial DNA
CM: chicken manure
MGB: minor groove binder
FAM: 6-carboxy-fluorescein
NFQ: non-fluorescent quencher
LOD: limits of detection
